# Preparation and Characterization of Chitosan/Soy Protein Isolate Nanocomposite Film Reinforced by Cu Nanoclusters

**DOI:** 10.3390/polym9070247

**Published:** 2017-06-25

**Authors:** Kuang Li, Shicun Jin, Xiaorong Liu, Hui Chen, Jing He, Jianzhang Li

**Affiliations:** 1 Key Laboratory of Wood Materials Science and Utilization, Beijing Forestry University, Ministry of Education, Beijing 100083, China; kuangli@bjfu.edu.cn (K.L.); jinsc1994@bjfu.edu.cn (S.J.); happyrong1993@bjfu.edu.cn (X.L.); 2 Beijing Key Laboratory of Wood Science and Engineering, Beijing Forestry University, Beijing 100083, China; 3 College of Materials Science and Technology, Beijing Forestry University, Beijing 100083, China

**Keywords:** soy protein isolate, Cu nanoclusters, chitosan, nanocomposite film, mechanical properties, water vapor barrier

## Abstract

Soy protein isolate (SPI) based films have received considerable attention for use in packaging materials. However, SPI-based films exhibit relatively poor mechanical properties and water resistance ability. To tackle these challenges, chitosan (CS) and endogenous Cu nanoclusters (NCs) capped with protein were proposed and designed to modify SPI-based films. Attenuated total reflectance-Fourier transform infrared spectroscopy and X-ray diffraction patterns of composite films demonstrated that interactions, such as hydrogen bonds in the film forming process, promoted the cross-linking of composite films. The surface microstructure of CS/SPI films modified with Cu NCs was more uniform and transmission electron microscopy (TEM) showed that uniform and discrete clusters were formed. Compared with untreated SPI films, the tensile strength and elongation at break of composite films were simultaneously improved by 118.78% and 74.93%, respectively. Moreover, these composite films also exhibited higher water contact angle and degradation temperature than that of pure SPI film. The water vapor permeation of the modified film also decreased. These improved properties of functional bio-polymers show great potential as food packaging materials.

## 1. Introduction

Polymer nanocomposites have emerged as an important research field in order to confer materials with superior properties [1,2]. Due to the advantages over conventional petroleum-based materials, researchers are committed to developing biopolymer-based films for use in packaging materials and coating industries [3,4]. Soy protein isolate (SPI) is a plant protein that is a reproducible resource, is safe, has good film-forming ability, and biocompatibility [5]. SPI-based films exhibit significant potential applications in the biosciences and biotechnology [6,7]. However, films obtained from unmodified SPI have some weaknesses in mechanical properties such as tensile strength and flexibility, which are critical issues for commercial applications [8]. Many traditional methods have been applied by previous studies, including different processing methods [9,10], blending organic or inorganic materials [11,12], and chemical cross-linking [13]. However, these efforts did not simultaneously improve the stiffness and flexibility of biopolymer films. González [14] reported that with the addition of starch nanocrystals, the tensile strength (TS) of SPI films could increase from 1.10 to 5.08 MPa, but the elongation at break (EB) of SPI film would decrease from 65.95% to 21.35%. Alipoormazandarani [4] reported that films modified with halloysite nanoclay showed enhanced TS, but poor EB. The reports of Jensen and Zhang also demonstrated that cellulose fibers or TiO_2_ improved the strength of films with a loss of flexibility [15,16]. Therefore, it is necessary to enhance both the strength and flexibility of SPI-based films simultaneously for further application.

As one of the most abundant natural biopolymers, chitosan is the *N*-deacetylated derivative of chitin and contains β-1-4 linked 2-amino-2-deoxy-d-glucopyranose repeat units [17,18]. Previous studies have shown that chitosan may interact with proteins to form films with enhanced mechanical properties [19,20,21]. However, natural defects and compatibility problems of the components might limit further application of these CS/SPI composite films. Meanwhile, functional nanomaterials have attracted much attention in recent decades as these nanoparticles could offer promising alternatives for improving polymer properties and extending their application fields [22,23]. Cu nanoclusters (NCs), composed of a few to hundreds of atoms, are defined as particles less than 2 nm and are considered to be novel potential nanomaterials [24,25]. The dimension of this nanoparticle approaches the Fermi wavelength of electrons. The discrete energy levels of electrons make the chemical, electronic and optical properties of this nanoparticle significantly different from conventional bulk materials [26,27]. In particular, Cu NCs exhibit good biocompatibility and multifunctional surface chemistry; thus, this nanomaterial may produce great results in improving the strength and compatibility of polymers [28].

Therefore, the objective of this work was to investigate the effects of chitosan and Cu NCs on the mechanical properties of SPI-based films. The modified SPI-based nanocomposite films were investigated by attenuated total reflectance-Fourier transform infrared (ATR FT-IR) spectroscopy, X-ray diffraction (XRD), contact angles determination, scanning electron microscopy (SEM), thermogravimetric analysis (TGA), moisture content (MC), and water vapor permeability (WVP). These characterizations indicated that CS and Cu NCs had a large influence on the structure, surface hydrophobicity, morphology, thermal stability, moisture content and water vapor permeability of SPI-based films, which are important properties of composite films in packaging material applications.

## 2. Materials and Methods

### 2.1. Materials

SPI with 2.0% moisture and 95% protein content was purchased by Yuwang Ecological Food Industry Co., Ltd. (Shandong, China). Copper sulfate anhydrous was obtained from Beijing Chemical Works (Beijing, China). Chitosan (95% deacetylation, biochemical reagent grade) was provided by Sinopharm Chemical Reagent Co., Ltd. (Beijing, China). Sodium hydroxide (analytical grade) was provided from Beijing Chemical Reagents (Beijing, China). Deionized water was used to prepare all aqueous solutions.

### 2.2. Preparation of SPI–Cu NCs

An amount of 4.0 g SPI was dissolved in 80 g distilled water with constant magnetic stirring (RW20 digital, IKA, Staufen, Germany) at 200 rpm to prepare the SPI solutions. Next, 8 mL CuSO_4_ solution (20 mmol/L) was added into these SPI solutions. The mixed solutions were stirred at 200 rpm at 25 °C for 10 min and adjusted pH to 12 with the NaOH solution. The mixture was heated under constant stirring at 75 °C for 8 h to prepare the SPI–Cu NCs solutions.

### 2.3. Preparation of CS/SPI–Cu NCs Composite Films

Chitosan solution (1%, *w*/*w*) was prepared by dissolving chitosan in acetic acid solution (2%, *v*/*v*) with continuous stirring at 200 rpm. Then, 8.0 g chitosan solution and 2.0 g glycerol was added into 80 g SPI–Cu NCs solution that was prepared before. According to the report of Jia et al. [29], chitosan was only soluble in acidic solution and the film prepared at pH 3.0 displayed better water barrier and mechanical properties. Therefore, the pH of the mixing solution was adjusted to 3.0. Next, the mixtures were stirred constantly at 200 rpm at 85 °C for 30 min and then poured into Teflon-coated plates. The films were vacuum-dried at 45 °C for 24 h. According to Schmid, M., et al. [30], all the films were tested at the same time, about a week after production, and the results were reproducible.

### 2.4. Characterization of SPI/CS Nanocomposite Films

#### 2.4.1. Transmission Electron Microscopy (TEM)

SPI–Cu NCs solutions were analyzed by TEM. SPI–Cu NCs solutions were poured on a copper wire mesh and dried in an oven at 45 °C for 30 min. Next, TEM images were observed on a JEM-1010 transmission electron microscopy (JEOL, Tokyo, Japan).

#### 2.4.2. Attenuated Total Reflectance-Fourier Transform Infrared Spectroscopy

Attenuated total reflectance-Fourier transform infrared (ATR-FTIR) spectra were performed by a Nicolet 6700 spectrometer (Thermo Scientific, Madison, WI, USA) with a range of 4000–650 cm^−1^ and 32 scans.

#### 2.4.3. X-Ray Diffraction Analysis

X-ray diffraction (XRD) was used by a D8 advance diffractometer (Bruker, Karlsruhe, Germany) with a radiation source of Cu–Ka. The data were measured from 5° to 60° with a 0.02° step interval at a voltage of 45 kV.

#### 2.4.4. Scanning Electron Microscope

Scanning electron microscopy (SU8010, Hitachi, Tokyo, Japan) was carried out to analyze the surface morphologies of composite films with an acceleration voltage of 5 kV.

#### 2.4.5. Mechanical Properties

Mechanical properties of the nanocomposite films were measured with a tensile testing machine (INSTRON 3365, INSTRON, Norwood, MA, USA) at a speed of 20 mm/min at 25 °C and a relative humidity of 50% as per the report of Li et al. [31]. Each film sample was cut into pieces with a size of 10 mm × 80 mm. Five specimens were measured to obtain the average values of tensile strength (TS), Young’s modulus (*E*) and elongation at break (EB).

#### 2.4.6. Surface Contact Angles

Surface hydrophobicity was characterized by water contact angle (OCA20, DataPhysics Instruments GmbH, Filderstadt, Germany). Prior to testing, all samples were conditioned in a saturated K_2_CO_3_-regulated desiccator at 50% ± 2% relative humidity and 25 °C. A drop of distilled water (3 μL) was dropped onto the surface of film samples (20 mm × 80 mm). Five replicates were tested for each film.

#### 2.4.7. Thermo-Gravimetric Analysis

The thermal stability of films was characterized by a Q50 TGA analyzer (TA Instrument, New Castle, DE, USA). Films were dried at 105 °C for 24 h and then heated from 10 °C to 600 °C at a rate of 10 °C/min in a nitrogen atmosphere (100 mL/min).

#### 2.4.8. Moisture Content

Five specimens were tested to calculate the moisture content of each film, and all films were conditioned in a saturated K_2_CO_3_-regulated (50% ± 2% relative humidity) desiccator at 25 °C before testing. Film specimens were weighted and recorded as m_0_, and then dried in an air-circulating oven at 105 °C for 24 h and weighed (*m*_d_). Moisture content for each film was calculated as follows:

MC (%) = (*m*_0_ − *m*_d_)/*m*_0_ × 100 (1)

#### 2.4.9. Water Vapor Permeability

Water vapor permeability was tested by a water vapor permeability tester (TSY-T1, Labthink Instrument, Jinan, China) as per the ASTM E96-01 standard method. Distilled water was poured into the test dish and placed in the controlled chamber at 25 °C and 50% relative humidity for 11 h. Five samples were tested for each film. The WVP was calculated as follows:

WVP = WVTR*x*/[*P*_0_(RH_1_ − RH_2_)] (2)

where WVTR is the measured water vapor transmission rate (g/m^2^ h) through each film, *x* is related to the thickness (mm) of the film; P_0_ is related to the vapor pressure of pure water (25 °C, 3.169 kPa); and (RH_1_ − RH_2_) is the relative humidity gradient used in the experiment which was controlled at 50% during the test.

#### 2.4.10. Statistical Analysis

Experiments were done in five replicates and data were analyzed by the analysis of variance (ANOVA) with the SPSS computer program. Tukey’s test was conducted to determine the post hoc multiple comparisons with the level of significance set at *p* < 0.05.

## 3. Results

### 3.1. Characterization of SPI–Cu NCs

**Figure 1:**
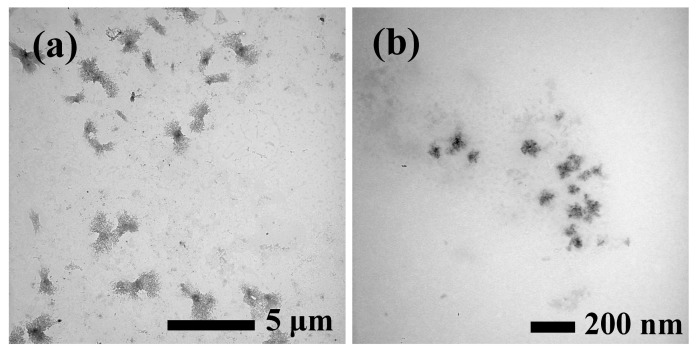
Transmission electron microscopy (TEM) images of soy protein isolate (SPI) based Cu nanoclusters (NCs) in solution (**a**) and (**b**).

The morphology and size of Cu NCs synthesized in the SPI substrate were characterized by transmission electron microscopy (TEM) images. As shown in Figure 1, Cu NCs were prepared by using an SPI template, which showed uniform and discrete clusters in the prepared solution. The average diameter of Cu NCs was approximately 50 nm (Figure 1b).

### 3.2. Structural Analysis

**Figure 2:**
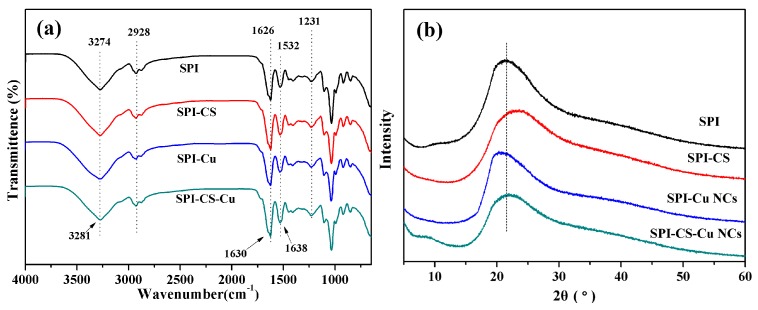
(**a**) Attenuated total reflectance-Fourier transform infrared (ATR-FTIR) spectra; and (**b**) X-ray diffraction (XRD) patterns of soy protein isolate (SPI) based films unmodified and modified with chitosan (CS) and Cu nanoclusters (NCs).

Attenuated total reflectance-Fourier transform infrared (ATR-FTIR) spectra were performed to investigate the changes of the functional groups in SPI-based film. As seen in Figure 2a, all SPI-based films exhibited similar spectra. The peaks at 1626, 1532 and 1231 cm^−1^ were assigned to amide I (C–O stretching), amide II (N–H bending) and amide III (C–H and N–H stretching), respectively [10]. Amide I and II of the SPI films treated by Cu NCs shifted to higher wavenumbers, which suggested the possible existence of hydrogen bonding between these polymers [32]. The peak at 2928 cm^−1^ appeared due to C–H stretching [2]. The broad absorption band observed around 3274 cm^−1^ was attributable to free and bound O–H and N–H groups, which formed hydrogen bonding with the carbonyl group of the peptide linkage in the protein matrix [5]. The increase in vibrational wavenumber of O–H and N–H vibration bands could be indicative of interactions between SPI and Cu NCs in composite film via hydrogen bonding [33]. These changes confirmed that the structures of SPI and chitosan were more unfolded and loose, which might make the films expose more polar and functional groups [34]. These interactions determined the mechanical properties and hydrophobicity of composite films, which were attributed to the effects of hydrogen bonds or electrostatic interactions between chitosan and Cu NCs.

X-ray diffraction (XRD) was conducted to investigate the changes of the structure of SPI-based films prepared with chitosan and Cu NCs. Figure 2b displays the XRD data of the SPI-based films untreated and treated with chitosan and Cu NCs. The XRD patterns of the SPI-based films were similar, and all films exhibited a relatively strong characteristic peak at 2θ = 22°, which represented the β-sheet structures of the SPI secondary structure [35]. The intensity of the peaks at 2θ = 22° of the modified films were slightly lower than that of the control film, due to the destruction of the SPI structure during the formation of nanocomposite films, indicating that Cu NCs changed the conformation of SPI and decreased the regular arrangement of the molecular chains. These results might make composite expose more active groups and prompt further reactions [36].

### 3.3. Micromorphology of SPI-Based Film

**Figure 3:**
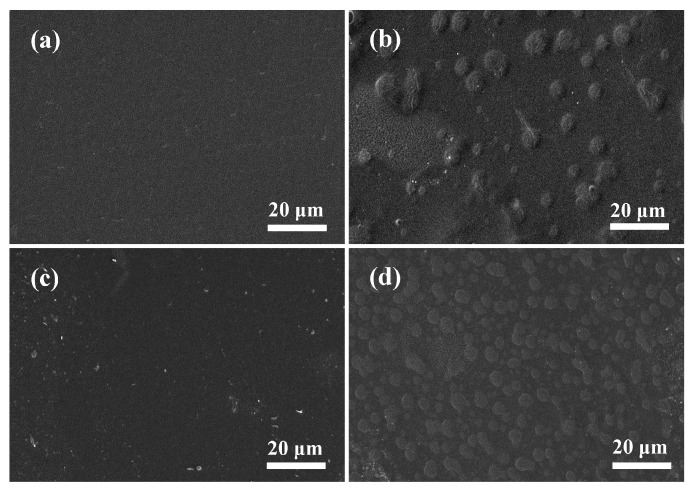
Scanning electron microscope (SEM) images of the surface of soy protein isolate (SPI) based films unmodified and modified with chitosan (CS) and Cu nanoclusters (NCs): (**a**) SPI; (**b**) SPI–CS; (**c**) SPI–Cu NCs; and (**d**) SPI–CS–Cu NCs.

SEM analysis was performed to investigate the microstructure of the composite films and the compatibility of the polymers. As seen in Figure 3a, the control film exhibited a smooth, compact and continuous surface, indicating that SPI had good film-forming ability. However, the images (Figure 3b) of SPI-based films modified with chitosan showed a relatively rougher surface morphology; there were some agglomerates on the surface of SPI–CS film, suggesting the formation of a heterogeneous structure. This phenomenon indicated the presence of thermodynamic incompatibility and phase segregation of SPI and chitosan [37]. The SPI–Cu NCs films showed a relatively coarse surface (Figure 3c), which may have been caused by the dispersion of Cu NCs. In particular, the surface of the CS/SPI film was more homogeneous (Figure 3d) with the incorporation of Cu NCs, and the agglomerates became more regular and uniform than those of the SPI–CS film. This change demonstrated that uniform dispersion of Cu NCs in the film matrix and Cu NCs may improve the phase compatibility of the blend components. SEM observations suggested that chitosan and Cu NCs had an important role in the organization of composite, which in turn determined the properties of SPI-based films. The compatibility of these polymers was more likely to enhance the molecular interactions in the SPI matrix, such as hydrogen bonding and electrostatic interactions, resulting in the improved mechanical and physical properties of the composited films.

### 3.4. Physical and Mechanical Properties

Mechanical properties of composite films are very important and determine the potential application of films as packaging materials. Tensile strength (TS), Young’s modulus (*E*), and elongation at break (EB) of SPI-based films were analyzed and are shown in Table 1.

Without a plasticizer, SPI-based film exhibited low strength and flexibility due to the rigid and brittle properties of protein. However, with the modification of chitosan, the TS values of SPI–CS films increased slightly, indicating that the addition of chitosan could improve the strength of SPI films slightly, which was attributed to the hydrogen bonding in the polymers [21]. Moreover, the results also showed that the EB values of composite films obviously increased from 17.63% to 62.86% with the modification of chitosan, indicating that chitosan might cause a plasticizing effect on the protein matrix [38]. Meanwhile, it was reported that intermolecular interactions were most likely established in these polymers, such as ionic and hydrophobic interactions [39]. Therefore, the flexibility of composite films was obviously improved.

Moreover, the TS and E values of SPI-based films modified with chitosan and Cu NCs showed an obvious increase with values of 5.01 MPa and 197.50 MPa, respectively. The significant enhancement of mechanical strength could be attributed to the effect of Cu NCs on the protein matrix and interfacial compatibility [40]; this result was supported by previous SEM analysis. Therefore, the structure of the composite film exhibited increased contact areas that promoted interactions in polymers, which resulted in a higher degree of molecular cross-linking and physically entangled structure in SPI–CS films. It is worth noting that compared with untreated SPI film, the TS and E values of SPI-based films modified with chitosan and Cu NCs were simultaneously increased, suggesting that the modification of chitosan and Cu NCs had a great effect on the reinforcement of the SPI-based films.

### 3.5. Contact Angles Analysis

**Figure 4:**
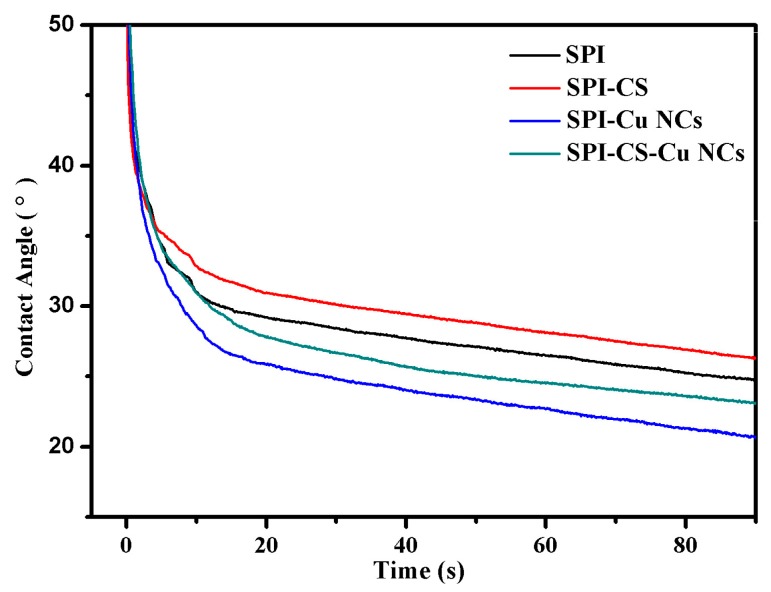
Water contact angles of soy protein isolate (SPI) based films unmodified and modified with chitosan (CS) and Cu nanoclusters (NCs).

The surface hydrophobicity was evaluated by measuring the contact angle of a water droplet on the surface of composite films. Generally, protein films possess high-water sensitivity, which is not desirable for packaging applications. Compared with the control film, the water contact angle of SPI–CS films increased, which could be attributed to the relatively high hydrophobic property of chitosan (Figure 4). Compared with the SPI film, the SPI–Cu NCs film exhibited a lower water contact angle, indicating a highly hydrophilic and wettable surface. This feature was due to the high content of polar groups on the surface of composite films [41]. These observations suggested that chitosan might result in the exposure of hydrophobic groups of soy proteins, thus increasing the surface hydrophobicity of films [29]. Therefore, the results demonstrated that SPI-based films modified with chitosan and Cu NCs exhibited preferable surface hydrophobicity and had great potential to overcome the limitation of hygroscopic property.

### 3.6. Thermo-Gravimetric Analysis

**Figure 5:**
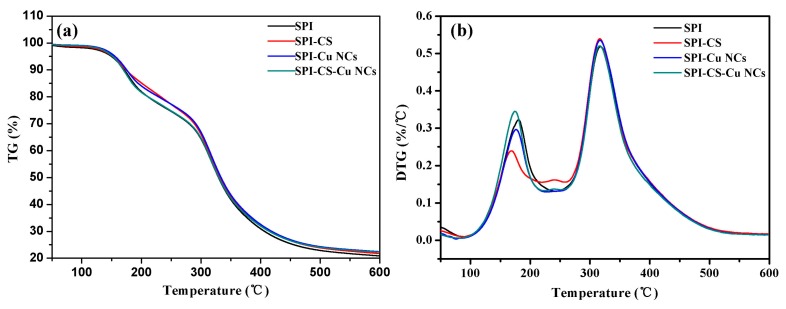
(**a**) Thermo gravimetric (TG) and (**b**) derivative thermo gravimetric (DTG) curves of soy protein isolate (SPI) based films unmodified and modified with chitosan (CS) and Cu nanoclusters (NCs).

The thermal stability of SPI-based films was investigated using thermo gravimetric (TG) and derivative thermo gravimetric (DTG), as shown in Figure 5. There were four steps to achieve the thermal degradation of composite films in the temperature range of 100–600 °C. The first step was from room temperature to 120 °C and corresponded to the loss of water in films. The second step of 120 °C to 280 °C was due to the degradation of glycerol. The degradation rate of films appeared between 280 °C and 450 °C, and was attributed to the decomposition of the SPI matrix. The last step was from 450 °C to 600 °C for the carbonized polymers in films [42].

These results suggested that the SPI–Cu films modified with chitosan showed a higher heat resistance and degradation temperature than the control film. It was reported that chitosan had a main weight loss at 300 °C, which might explain why the composite films exhibited lower rates of decomposition below 300 °C. Therefore, the modification of chitosan and Cu NCs might improve the thermal stability of SPI-based films.

### 3.7. Water Resistance

Moisture content (MC) was related to the water resistance ability of films. Table 2 shows the moisture content for the control film and the SPI-based films with chitosan and Cu NCs. Compared with the control film, the SPI based films modified with chitosan (CS) and Cu nanoclusters (NCs) presented an obvious increase in the value of MC, which was due to the structural changes of the films made by the swelling of the hydrophilic fraction of the polymer matrix [29].

Meanwhile, water vapor barrier properties were also investigated by water vapor permeation (WVP). The resistance of the films against water vapor was highly associated with the micro-paths in the network microstructures of the SPI matrix. Without modification, the water vapor permeability of the SPI-based films was poor due to the hydrophilicity of the protein molecule. As shown in Table 2, the WVP of the SPI–CS film was higher than that of the control film. This change might have resulted from the phase separation between the SPI and chitosan during preparation, and the addition of chitosan to the SPI solution might decrease the intermolecular interactions (such as hydrogen binding) and increase the electrostatic repulsion between these two polymers [39]. Compared with the SPI and SPI–CS films, the WVP of SPI-based films modified with Cu NCs was significantly decreased, indicating a better water vapor barrier property of the composite films. The lower WVP of the composite films modified with Cu NCs could be due to the improved cross-linking of the SPI chains as well as the strong interactions between polymers [43,44]. With the modification of Cu NCs, the compatibility of SPI and chitosan molecules might be improved, which resulted in increased circuitous routes of films [17].

## 4. Conclusions

In summary, we synthesized water-dispersed Cu NCs capped with the soy protein isolate with a facile method, and used Cu NCs and chitosan to modify the mechanical properties of SPI-based films. Compared with unmodified films, the modification of chitosan and Cu NCs improved the strength and flexibility of the composite films where the TS and EB values of the SPI–CS–Cu NCs films increased by 118.78% and 74.93%, respectively, which might have been caused by the interactions in the SPI matrix. Compared with the TS of soybean protein film [11], the TS of SPI–CS–Cu NCs films significantly increased from 3.54 to 5.01 MPa, suggesting the reinforced mechanical property. The addition of Cu NCs improved the compatibility between the SPI and chitosan, thus the microstructures of the SPI-based films were more uniform. Meanwhile, with the modification of chitosan and Cu NCs, SPI-based films also showed a higher water contact angle and degradation temperature, suggesting better hydrophobicity and thermal stability of the composite films. Compared with isolated soy protein film [45], SPI–CS–Cu NCs film also led to a decrease in WVP, indicating superior water vapor barrier ability. The improved functional properties of this novel nanocomposite film contributed to its potential application in food packaging materials.

## Figures and Tables

**Table 1:** Tensile strength (TS), Young’s modulus (*E*) and elongation at break (EB) of soy protein isolate (SPI) based films unmodified and modified with chitosan (CS) and Cu nanoclusters (NCs).

Samples	Thickness	TS	*E*	EB
	(mm)	(MPa)	(MPa)	(%)
SPI	0.213 (0.019) ^b^	2.29 (0.16) ^c^	58.75 (2.52) ^c^	17.63 (0.09) ^c^
SPI–CS	0.190 (0.024) ^c^	3.02 (0.28) ^b^	67.89 (3.29) ^c^	62.86 (0.04) ^a^
SPI–Cu NCs	0.234 (0.015) ^a^	3.55 (0.21) ^b^	149.20 (3.40) ^b^	17.05 (0.17) ^c^
SPI–CS–Cu NCs	0.240 (0.026) ^a^	5.01 (0.34) ^a^	197.50 (4.05) ^a^	30.84 (0.13) ^b^

The values in parenthesis are the standard deviation, ^a–c^ Two means in the same column followed by the same letter are not significantly (*p* > 0.05) different through the Tukey’s multiple range test.

**Table 2:** Moisture content (MC) and water vapor permeation (WVP) of soy protein isolate (SPI) based films unmodified and modified with chitosan (CS) and Cu nanoclusters (NCs).

Samples	Moisture content (%)	Water vapor permeation (g·mm·h^−1^·m^−2^·kPa^−1^)
SPI	9.89 (1.3) ^c^	1.16 (0.15) ^b^
SPI–CS	15.40 (1.6) ^a^	1.27 (0.19) ^a^
SPI–Cu NCs	11.84 (1.1) ^b^	0.99 (0.12) ^c^
SPI–CS–Cu NCs	14.09 (1.8) ^a^	1.10 (0.16) ^b^

The values in parenthesis are the standard deviations, ^a–c^ Two means in the same column followed by the same letter are not significantly (*p* > 0.05) different through the Tukey’s multiple range test.
